# Pituitary Dysfunction after Blast Traumatic Brain Injury: The UK BIOSAP Study

**DOI:** 10.1002/ana.23958

**Published:** 2013-09-24

**Authors:** David Baxter, David J Sharp, Claire Feeney, Debbie Papadopoulou, Timothy E Ham, Sagar Jilka, Peter J Hellyer, Maneesh C Patel, Alexander N Bennett, Alan Mistlin, Emer McGilloway, Mark Midwinter, Anthony P Goldstone

**Affiliations:** 1 Computational Cognitive and Clinical Neuroimaging Laboratory, Division of Brain Sciences Imperial College London, Hammersmith HospitalLondon; 2 Royal Centre for Defence Medicine, Academic Department of Military Surgery and TraumaBirmingham; 3 Imperial Centre for Endocrinology, Imperial College Healthcare NHS Trust, Charing Cross HospitalLondon; 4 Imaging Department, Imperial College Healthcare NHS Trust, Charing Cross HospitalLondon; 5 Defence Medical Rehabilitation CentreHeadley Court, Epsom, Surrey; 6 Academic Section for Musculoskeletal Disease, Chapel Allerton Hospital, University of LeedsLeeds; 7 Metabolic and Molecular Imaging Group, Medical Research Council Clinical Sciences Centre Imperial College London, Hammersmith HospitalLondon, United Kingdom

## Abstract

**Objective:**

Pituitary dysfunction is a recognized consequence of traumatic brain injury (TBI) that causes cognitive, psychological, and metabolic impairment. Hormone replacement offers a therapeutic opportunity. Blast TBI (bTBI) from improvised explosive devices is commonly seen in soldiers returning from recent conflicts. We investigated: (1) the prevalence and consequences of pituitary dysfunction following moderate to severe bTBI and (2) whether it is associated with particular patterns of brain injury.

**Methods:**

Nineteen male soldiers with moderate to severe bTBI (median age = 28.3 years) and 39 male controls with moderate to severe nonblast TBI (nbTBI; median age = 32.3 years) underwent full dynamic endocrine assessment between 2 and 48 months after injury. In addition, soldiers had structural brain magnetic resonance imaging, including diffusion tensor imaging (DTI), and cognitive assessment.

**Results:**

Six of 19 (32.0%) soldiers with bTBI, but only 1 of 39 (2.6%) nbTBI controls, had anterior pituitary dysfunction (*p* = 0.004). Two soldiers had hyperprolactinemia, 2 had growth hormone (GH) deficiency, 1 had adrenocorticotropic hormone (ACTH) deficiency, and 1 had combined GH/ACTH/gonadotrophin deficiency. DTI measures of white matter structure showed greater traumatic axonal injury in the cerebellum and corpus callosum in those soldiers with pituitary dysfunction than in those without. Soldiers with pituitary dysfunction after bTBI also had a higher prevalence of skull/facial fractures and worse cognitive function. Four soldiers (21.1%) commenced hormone replacement(s) for hypopituitarism.

**Interpretation:**

We reveal a high prevalence of anterior pituitary dysfunction in soldiers suffering moderate to severe bTBI, which was more frequent than in a matched group of civilian moderate to severe nbTBI subjects. We recommend that all patients with moderate to severe bTBI should routinely have comprehensive assessment of endocrine function. Ann Neurol 2013;74:527–536

The use of improvised explosive devices (IEDs) has characterized the Iraq and Afghanistan conflicts, with blast traumatic brain injury (bTBI) a “signature injury.”^1^ More than 400 UK and 2,000 US soldiers have been fatally wounded by blast injuries in Afghanistan since 2001.^2^ Among survivors, it is estimated that 19.5% of 1.64 million US troops deployed in both conflicts have suffered a probable bTBI.^3^ Soldiers are usually young, so the long-term impact of consequent physical, cognitive, and psychological problems represents a significant health burden. There are no current pharmaceutical treatments that improve recovery following TBI.^4^

Nonblast TBI (nbTBI) is a recognized cause of pituitary dysfunction, in particular growth hormone (GH) deficiency.^5^ Reported prevalence rates of pituitary dysfunction following nbTBI vary between 2 and 68%.^5^,^6^ This variability is due in part to differences in the normal ranges and dynamic endocrine tests used, the time since injury, and injury severity.^5^–^7^ In addition to adverse metabolic consequences, hypopituitarism causes multiple symptoms impacting on physical and psychological well-being that will impair recovery after TBI, and thus hormone replacement represents an important therapeutic opportunity.^8^–^11^ It is unknown how often bTBI leads to pituitary dysfunction.^12^

Diffusion tensor imaging (DTI) is a sensitive magnetic resonance (MR) technique that can assess the presence and severity of white matter damage after TBI.^13^,^14^ TBI alters the pattern of water diffusion within white matter, resulting in abnormal diffusion measures, including fractional anisotropy (FA). DTI abnormalities in several brain regions have been reported in soldiers following mild bTBI.^15^ We hypothesized that DTI would reveal differences in white matter damage in those soldiers with pituitary dysfunction after bTBI.

Here we report findings from the UK BIOSAP (United Kingdom Blast Injury Outcome Study of Armed Forces Personnel). We investigated the prevalence and associations of pituitary dysfunction in soldiers after moderate to severe bTBI compared to a control group of patients after nbTBI.

## Subjects and Methods

### Recruitment

Nineteen military bTBI patients were recruited using the Academic Department of Military Emergency Medicine (Birmingham, UK) trauma database to identify soldiers injured between December 2009 and March 2012. This represents 10.4% of the 183 UK soldiers who had survived a moderate to severe bTBI in Afghanistan during this 27-month period, of what is now the 12th year of this conflict. Research ethics committee approval and informed consent were obtained.

Comparison was made with an age- and gender-matched control group of 39 patients after nbTBI. This represented all the patients seen in our multidisciplinary Traumatic Brain Injury clinic at Charing Cross Hospital, London, United Kingdom between August 2009 and March 2012 who met all inclusion/exclusion criteria and were within the age range of the bTBI group. These patients had identical endocrine assessment as part of their routine clinical care.

The inclusion criterion for bTBI was a moderate to severe brain injury caused directly by a single exposure to a blast. To better examine the effects of the primary blast wave only, exclusion criteria for bTBI were: (1) requirement for massive blood transfusion; (2) intracranial lesions causing mass effect; and (3) post-traumatic stress disorder (PTSD), because this has been linked with endocrine disturbance.^16^,^17^ PTSD was diagnosed on the basis of psychologist interview and, if suspected, subsequent self-reported symptom ratings from the PTSD Checklist–Military version derived from Diagnostic and Statistical Manual of Mental Disorders, 4th edition criteria.^18^ Although this includes symptoms present in many soldiers after bTBI, such as loss of memory of the event, anhedonia, social isolation, sleep disturbance, emotional lability, and poor concentration, subjects did not display additional symptoms required for the diagnosis of PTSD, such as “repeated, disturbing memories, thoughts, images or dreams of a previous stressful experience” or “physical reactions (such as heart pounding, trouble breathing or sweating) when reminded of a previous stressful experience.”

Inclusion criteria for both bTBI and nbTBI were: (1) male gender, (2) >2 and <48 months from a single TBI, (3) moderate to severe brain injury using the Mayo classification criteria,^19^ (4) ongoing cognitive and/or psychological symptoms, and (5) completion of all endocrine testing. Exclusion criteria for bTBI and nbTBI subjects were: (1) diabetes mellitus, (2) pre-TBI history of psychiatric disorder, (3) current or previous drug or excess alcohol use, (4) reversed sleep–wake cycle, and (4) craniotomy following injury (to avoid the difficulties in brain image registration resulting from gross changes in brain structure).

Both bTBI and nbTBI subjects underwent clinical assessment and calculation of Abbreviated Injury Score (AIS) and total Injury Severity Score (ISS), and completed quality of life (QoL) and symptom questionnaires (see Supplementary Methods).

### Endocrine Testing

The algorithm used to define pituitary dysfunction is shown in Table 1 (see Supplementary Methods). All patients had measurement of basal serum anterior pituitary hormones followed by dynamic endocrine testing. Initial screening for GH and adrenocorticotropic hormone (ACTH) deficiency used the glucagon stimulation test (GST).^20^,^21^ The diagnosis of GH deficiency was confirmed with second-line growth hormone-releasing hormone (GHRH)–arginine test and/or insulin tolerance test (ITT).^10^,^22^,^23^ ACTH deficiency was confirmed with an ITT or metyrapone stimulation test, together with a cortisol day curve.^21^,^24^ Symptoms of diabetes insipidus were investigated further with a water deprivation test.

**Table 1:** Diagnostic Algorithm for Pituitary Dysfunction

Pituitary Axis	First Test	Confirmatory Test
GH deficiency	Glucagon stimulation test: peak GH < 5μg/l	GHRH–arginine test: GH < cutoff based on age and BMI;^22^ OR ITT: peak GH < 3μg/l
ACTH deficiency	Glucagon stimulation test: peak cortisol < 350nmol/l (<12.7μg/dl)^21^	Metyrapone test: 11-DOC < 200nmol/l (<6.9μg/dl) OR if unavailable ACTH < 60ng/l despite cortisol < 200nmol/l (<7.2μg/dl); OR ITT: peak cortisol < 450nmol/l (<16.3μg/dl); supported by am cortisol < 100nmol/l (<3.62μg/dl)
Hyperprolactinemia	Prolactin > 375 mU/l (NR = 75–375)	Repeat prolactin > 375mU/l AND negative macroprolactin AND normal MRI pituitary with contrast
Gonadotrophin deficiency	Random testosterone < 10nmol/l (<2.9ng/ml) OR if SHBG low (<15nmol/l) FAI < 30; AND nonelevated LH (NR = 1.7–12.0 IU/l) and FSH (NR = 1.7–8.0 IU/l)	Repeat abnormal basal levels using morning (9–10 am) sample
TSH deficiency	Free T4 < 9.0pmol/l (<0.70ng/dl) OR free T3 < 2.5pmol/l (<0.16ng/dl); AND nonelevated TSH (NR = 0.30–4.22mU/l)	Repeat abnormal basal levels
ADH (vasopressin) deficiency (diabetes insipidus)	Symptoms of polyuria or polydipsia AND random urine osmolarity <750 mosmol/kg	Water deprivation test

11-DOC = 11-deoxycorticosterone; ACTH = adrenocorticotropic hormone; ADH = antidiuretic hormone; BMI = body mass index; FAI = free androgen index (100 × testosterone/SHBG); FSH = follicle-stimulating hormone; GH = growth hormone; GHRH = growth hormone-releasing hormone; ITT = insulin tolerance test; LH = luteinizing hormone; MRI = magnetic resonance imaging; NR = normal range; SHBG = sex hormone-binding globulin; TSH = thyroid-stimulating hormone.

### Cognitive Function Assessment

Each soldier with bTBI completed a standardized neuropsychological test battery previously shown to be sensitive to cognitive impairment after TBI.^14^ The tests looked at the cognitive domains of: (1) current verbal and nonverbal reasoning ability; (2) associative memory and learning; (3) executive functions of set shifting, inhibitory control, cognitive flexibility, and word generation fluency; and (4) information processing speed (see Supplementary Methods).

### Structural Brain Imaging

Each soldier had standard T1, gradient-echo (T2*), and susceptibility-weighted MR imaging (MRI) to assess focal brain injury, microbleeds, superficial siderosis, gliosis, contusions, and DTI. Most patients with pituitary dysfunction also had a pituitary MRI with gadolinium contrast to look for more detailed hypothalamic–pituitary abnormalities. Patients with nbTBI had only computed tomography (CT) brain and/or standard T1/T2 brain MRI as part of routine clinical practice. DTI analysis of white matter tracts combined tract-based spatial statistics and region of interest (ROI) approaches (Functional Magnetic Resonance Imaging of the Brain Software Library, Oxford, UK), focusing on regions previously shown to be sensitive to damage in bTBI and nbTBI (Supplementary Fig S1 and Supplementary Methods).^14^,^15^ This allowed assessment of regional FA, a measure of traumatic axonal injury.

### Statistical Analyses

Comparisons between groups (nbTBI vs bTBI; and bTBI with pituitary dysfunction vs bTBI without pituitary dysfunction) were made using Fisher exact test for prevalence data, and unpaired Student *t* test (FA and neurocognitive variables), or Mann–Whitney *U* test (other variables) for continuous data (SPSS v19.0; IBM, Armonk, NY). Significance was defined as *p* < 0.05. A group × ROI repeated measure analysis of variance was performed to assess the overall effect of pituitary dysfunction on FA.

## Results

### Patient Characteristics

All soldiers with bTBI had been injured by IEDs and had been wearing full personal protective equipment. All required immediate transfer to Camp Bastion for emergency medical treatment, and repatriation to the United Kingdom within 48 hours. We have detailed information about the blast exposure, but for operational security reasons these cannot be reported. In the control nbTBI group, injuries were secondary to road traffic accidents (RTAs; 43%), assaults (32%), falls (23%), and sporting injuries (2%). Three subjects in the nbTBI group had experienced previous TBI (1 subject had 2 mild TBIs from an RTA and an assault, 1 a mild TBI from a fall, and 1 a TBI of unknown severity from an assault).

The bTBI and nbTBI groups were well matched in most respects (Table 2). There were no significant differences in age, ISS whole body injury severity, skull/facial fractures (15.8 vs 15.4%), or post-traumatic seizures (10.5 vs 7.7%). The bTBI group had longer post-traumatic amnesia (PTA; median 5.5 days vs 0.5 days, *p* = 0.01); more injuries requiring surgery to or loss of function of major extracranial organs (57.9 vs 7.7%, *p* = 0.002); more amputations (36.8 vs 0%, *p* < 0.001); and, in keeping with this, more use of strong prescription opiates (47.3 vs 7.7%, *p* = 0.001). The time from TBI to endocrine testing was significantly longer in the bTBI group (median 15.2 vs 5.8 months, *p* = 0.001).

**Table 2:** Patient Characteristics

Characteristic	Maximum Score	All nbTBI	All bTBI	*p*	bTBI: No Pituitary Dysfunction	bTBI: Pituitary Dysfunction	*p*
No.		39	19		13	6	
Age at TBI, yr		31.3 [22.5–35.7]	26.7 [26.1–30.9]	0.40	26.6 [24.6–30.6]	29.3 [25.8–36.6]	0.48
		17.2–44.8	19.0–43.5		19.0–36.3	25.0–43.5	
Age at testing, yr		32.3 [23.1–36.7]	28.3 [26.8–32.2]	0.40	28.0 [25.3–31.4]	30.3 [27.4–38.3]	0.32
		19.9–45.1	19.6–44.7		19.6–37.6	26.3–44.7	
Time since TBI, mo		5.8 [3.1–11.0]	15.2 [10.8–19.3]	0.001^a^	15.2 [8.8–16.6]	17.6 [12.3–20.2]	0.32
		1.9–41.2	4.1–23.7		4.1–23.7	4.9–21.9	
ISS	75	25.0 [16.0–32.0] 1–75	33.0 [20.0–45.0] 9–70	0.17	24.0 [14.5–40.5] 9–45	35.5 [27.0–51.3] 9–70	0.24
AIS head	6	5.0 [4.0–5.0] 1–6	4.0 [3.0–5.0] 0–6	0.04^a^	4.0 [2.5–4.0] 0–5	5.0 [3.0–5.3] 0–6	0.06
AIS chest	6	0 [0–0] 0–6	0 [0–2] 0–4	0.11	0 [0–3] 0–4	0.5 [0–2.3] 0–3	0.83
AIS abdomen	6	0 [0–0] 0–3	0 [0–2] 0–3	0.02^a^	0 [0–2] 0–2	0 [0–2.3] 0–3	0.97
GCS	15	14.0 [6.0–14.0]^b^ 3–15	3.0 [3.0–14.5]^c^ 3–15	0.24	14.0 [3.0–15.0]^d^ 3–15	3.0 [3.0–3.0]^e^ 3–3	0.19
PTA, days		0.5 [0–7.3]^f^ 0–42	5.5 [0.8–22.8] 0–84	0.01^a^	3.0 [0–19.3] 0–84	15.5 [6.3–31.5] 4–42	0.10
PTA > 24 hours		20 (51.3%)	13 (68.4%)	0.27	7 (58.3%)	6 (100%)	0.11
BMI, kg/m^2^		24.7 [22.4–29.4] 17.0–33.4	26.7 [24.5–28.9] 21.7–33.7	0.28	26.6 [24.5–28.7]^g^ 23.6–29.4	25.5 [22.4–32.0]^h^ 21.7–33.7	0.79
Limb amputation		0 (0%)	8 (42.1%)	<0.001^a^	6 (46.1%)	2 (33.3%)	1.00
Major organ damage		3 (7.7%)	11 (57.9%)	<0.001^a^	7 (53.9%)	4 (66.7%)	1.00
Skull/facial fracture		6 (15.4%)	3 (15.8%)	1.00	0 (0%)	3 (50.0%)	0.02
Opiate use		3 (7.7%)	9 (47.3%)	0.001^a^	6 (46.2%)	3 (50.0%)	1.00
Antidepressant use		5 (12.8%)^i^	10 (52.7%)^j^	0.003^a^	7 (53.8%)^k^	3 (50.0%)^l^	1.00
Seizures post-TBI		3 (7.7%)^m^	2 (10.5%)^n^	1.00	1 (7.7%)^o^	1 (16.7%)^p^	1.00
Primary hypogonadism		1 (2.6%)^q^	4 (21.1%)^r^	0.04^a^	4 (30.8%)^r^	0 (0%)^r^	0.26

Data are expressed as median [interquartile range], range, or No. (%). Probability values are from Mann–Whitney *U* test or Fisher exact test between groups.

^a^Statistically significant; *p* < 0.05.

Data available for ^b^n = 16, ^c^n = 9, ^d^n = 5, ^e^n = 4, ^f^n = 38, and due to amputations: ^g^n = 7, ^h^n = 4.

For analgesic purposes only in: ^i^n = 5 (12.8%), ^j^n = 6 (31.6%), ^k^n = 4 (30.8%), ^l^n = 2 (33.3%).

For depression itself in: ^i^n = 0 (0%), ^j^n = 4 (21.1%), ^k^n = 3 (23.1%), ^l^n = 1 (16.7%).

On antiepileptic drugs in ^m^n = 3, ^n^n = 1, ^o^n = 0, ^p^n = 1.

^q^Not due to trauma.

^r^Due to perineal trauma.

AIS = Abbreviated Injury Score; BMI = body mass index; bTBI = blast traumatic brain injury; GCS = Glasgow Coma Scale; ISS = Injury Severity Score; nbTBI = nonblast TBI; PTA = post-traumatic amnesia.

### Prevalence of Pituitary Function in bTBI and nbTBI Cohorts

**FIGURE 1:**
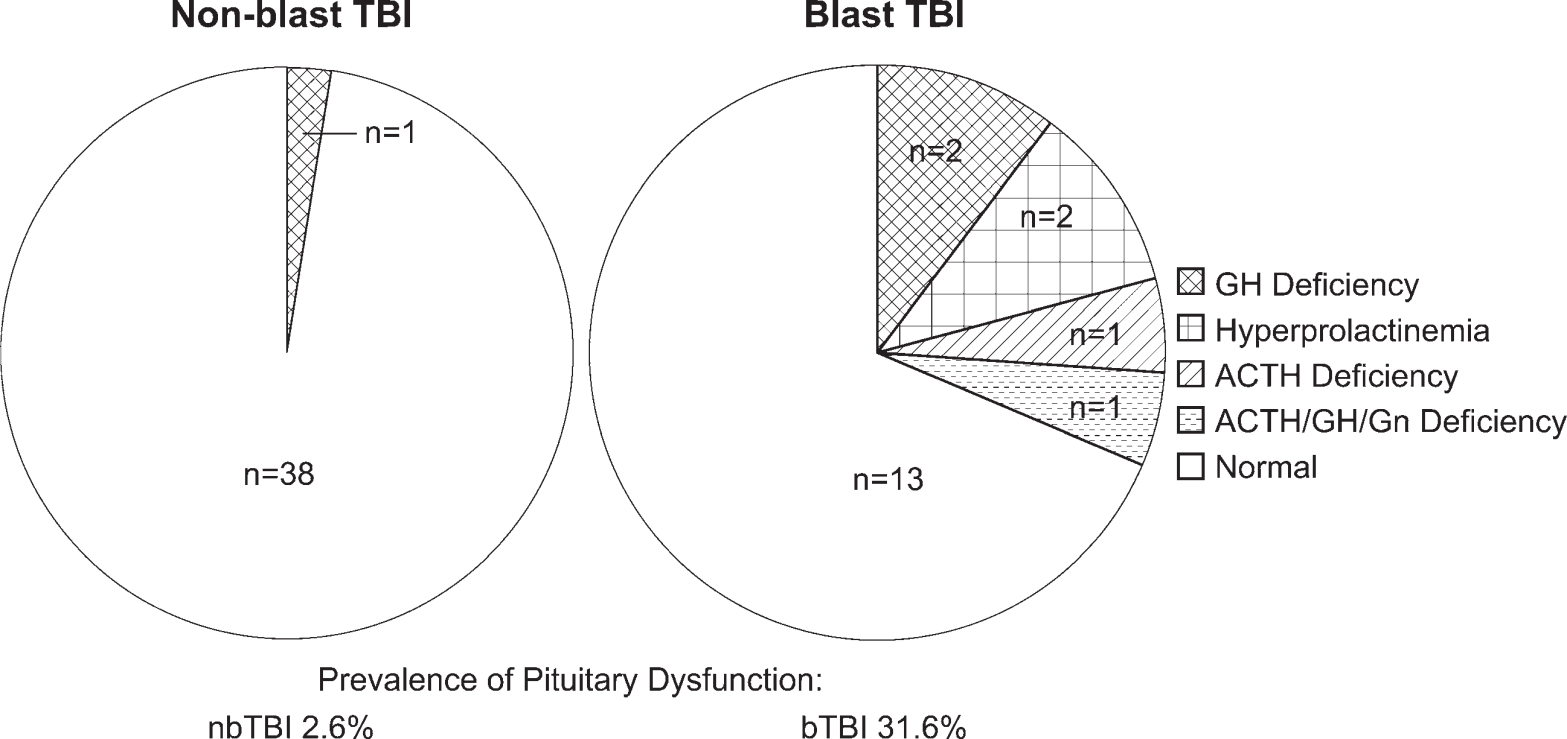
Prevalence of pituitary dysfunction in nonblast traumatic brain injury (nbTBI) and blast TBI (bTBI). Greater prevalence of anterior pituitary dysfunction was seen in subjects after bTBI (right) than nbTBI (left). No subjects had thyroid-stimulating hormone deficiency or diabetes insipidus. ACTH = adrenocorticotropic hormone; GH = growth hormone; Gn = gonadotrophin.

Six of 19 soldiers with bTBI (31.6%) had anterior pituitary dysfunction, compared to only 1 of 39 (2.6%) subjects with nbTBI (*p* = 0.004; Fig 1, Supplementary Tables S1–S3). Two soldiers (10.5%) had monomeric hyperprolactinemia (without secondary hypogonadism), 1 (5.3%) had isolated ACTH deficiency, 2 (10.5%) had isolated GH deficiency, and 1 (5.3%) had combined ACTH, GH, and gonadotrophin deficiencies. The only pituitary dysfunction noted in 1 patient with nbTBI was isolated GH deficiency following a single TBI. No patients in either group had thyroid-stimulating hormone (TSH) deficiency or diabetes insipidus.

The 3 soldiers with GH deficiency had insulin-like growth factor-I (IGF-I) levels in the low normal range (see Supplementary Table S2), and the 2 soldiers with ACTH deficiency had normal early morning cortisol levels on initial assessment of 287 to 292nmol/l equivalent to 10.3 to 10.5μg/dl (normal, >150nmol/l, >5.4μg/dl, respectively; see Supplementary Table S3). However, on subsequent cortisol day curves, both subjects with ACTH deficiency had low cortisol levels (<100nmol/l, 3.62μg/dl) at either 9:00 AM or 12:00 pm on a day curve consistent with the diagnosis (see Supplementary Results, Supplementary Table S3). Thus, although the less commonly used metyrapone test was occasionally performed as the confirmatory test to diagnose or exclude ACTH deficiency instead of the gold standard ITT, findings were always compatible with the results of baseline or day curve cortisol levels. Furthermore, as with previous studies, we have found good specificity and concordance between the results of the metyrapone test compared to the ITT or ACTH stimulation test for diagnosing ACTH deficiency (see Supplementary Results). None of the soldiers with ACTH deficiency had any history of hypotension, hypoglycemia, or hyponatremia.

Primary hypogonadism due to perineal/testicular blast injury had been found in an additional 4 of 19 soldiers with bTBI (21.2%), none of whom had pituitary dysfunction, and all were already on testosterone replacement (see Supplementary Results, Supplementary Table S1).

### Comparison of bTBI with versus bTBI without Pituitary Dysfunction

There was no significant difference in age at TBI, time since injury, ISS, abdominal AIS, body mass index (BMI), or prevalence of amputations, nonhead major organ damage, seizures, any use of antidepressants or specifically for depression, or opiate use between bTBI patients with versus those without pituitary dysfunction (see Table 2, Supplementary Tables S6 and S7). BMI could not be adequately assessed in the 8 soldiers with bTBI who had limb amputations, but none was morbidly obese on clinical examination.

There were trends for the AIS head injury scores to be higher (*p* = 0.06), and duration of PTA to be longer (median = 15.5 vs 3.0 days, *p* = 0.10) in those soldiers with pituitary dysfunction after bTBI than in those without.

The single soldier (M08) with multiple pituitary deficiencies was taking opiates at the time of diagnosis of gonadotrophin deficiency and initial dynamic endocrine testing with a GST. However, both GH and ACTH deficiency were subsequently confirmed using an ITT after opiates had been discontinued.

### Neuroimaging Results

In the bTBI group, we investigated whether particular structural abnormalities were associated with pituitary dysfunction. Three of the 6 (50.0%) soldiers with pituitary dysfunction, compared to only 1 of the 13 (7.7%) soldiers without pituitary dysfunction, had contusions on brain MRI scans (*p* = 0.07). One soldier with pituitary dysfunction had 2 contusions, whereas the remainder had 1 contusion (Supplementary Fig S2). The total contusion volume was <10cm^3^ in all cases; the soldier without pituitary dysfunction had the smallest contusion volume. There was a greater prevalence of skull/facial fractures in the soldiers with pituitary dysfunction compared to those without (50 vs 0%, *p* = 0.02).

There were no significant differences in the prevalence of other abnormalities visible on acute CT brain scans following blast exposure or study structural MR scans, including presence of extracerebral, subarachnoid, or intraventricular hemorrhage, microbleeds, superficial siderosis, or gliosis, between those soldiers with versus without pituitary dysfunction (Supplementary Table S4). No hypothalamic–pituitary abnormalities were seen on MRI brain scans in any soldiers in the bTBI group, or in the 4 with pituitary dysfunction who had dedicated contrast-enhanced MRI pituitary scans (M01, M08, M10, M14). This included all those soldiers with hyperprolactinemia and multiple pituitary hormone deficiencies.

**FIGURE 2:**
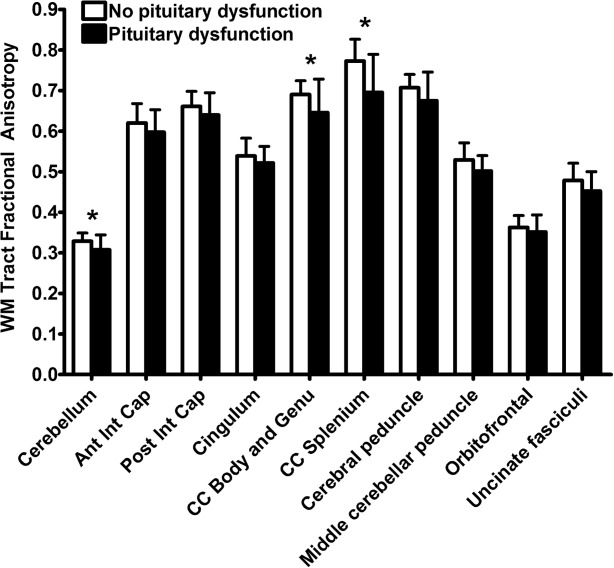
Pituitary dysfunction and white matter damage in blast traumatic brain injury. Lower fractional anisotropy was seen in a priori white matter tract regions of interest in soldiers with pituitary dysfunction after blast traumatic brain injury (black, n = 6) compared to those without pituitary dysfunction (white, n = 13). Data are expressed as mean ± standard deviation. *p < 0.05 (unpaired *t* test). Ant = anterior; CC = corpus callosum; Cap = capsule; Int = internal; Post = posterior; WM = white matter.

DTI analysis showed a reduction in FA depending on the ROI, indicating greater white matter damage, in those soldiers with pituitary dysfunction after bTBI compared to those without (*p* = 0.14 effect of group, *p* = 0.02 group × ROI interaction). Planned post hoc analysis showed significantly lower FA values for those soldiers with pituitary dysfunction within the cerebellum (*p* < 0.05), and body/genu (*p* < 0.05) and splenium (*p* = 0.01) of the corpus callosum ( Fig 2).

### Symptoms, QoL, and Cognitive Function

Consistent with their higher prevalence of polytrauma and amputations, the soldiers with bTBI had significantly worse scores for physical activity and daily living problems than the control nbTBI group, but not in measures of depression and emotional well-being (see Supplementary Table S5, Supplementary Results).

In the bTBI group, soldiers with pituitary dysfunction had trends toward worse measures of QoL and symptom scores in several domains relating to emotional and social functioning, fatigue, and mood compared to those without pituitary dysfunction (see Supplementary Table S5, Supplementary Results).

The bTBI subjects with pituitary dysfunction had significantly worse average current verbal intellectual ability than those without pituitary dysfunction, despite there being no significant difference in their premorbid intelligence (Wechsler Test of Adult Reading; Table 3). The bTBI group with pituitary dysfunction also showed significantly worse cognitive impairment in the domains of visual/naming/reading processing speed, verbal fluency, and information processing (see Table 3).

**Table 3:** Pituitary Dysfunction and Cognitive Function in Blast Traumatic Brain Injury

Cognitive Domain	Cognitive Variable	No Pituitary Dysfunction, n = 13	Pituitary Dysfunction, n = 6
Premorbid intelligence: reading ability	WTAR raw score	35.9 ± 11.7	34.7 ± 14.6
Intellectual ability	WASI similarities (verbal)	32.6 ± 6.2	27.0 ± 4.1^a^
	WASI matrix reasoning (nonverbal)	24.4 ± 7.5	24.2 ± 6.0
Memory: associative memory	People test immediate recall	22.6 ± 8.1	25.0 ± 7.8
Processing speed: visual search/complex	Trail Making Test trail A, seconds	23.1 ± 5.7	28.7 ± 5.2^a^
	Trail Making Test trail B, seconds	47.9 ± 14.5	53.8 ± 12.2
Processing speed: naming/reading	Stroop color naming, seconds	32.5 ± 9.1	51.0 ± 29.7^a^
	Stroop word reading, seconds	24.3 ± 6.7	37.2 ± 13.6^b^
Executive function: alternating-switch cost	Trail Making Test trail B − A, seconds	24.8 ± 13.5	25.2 ± 9.0
Executive function: cognitive flexibility	Color word Stroop inhibition/switching, seconds	70.5 ± 24.2	86.3 ± 30.8
	Inhibition/switching minus a baseline of color naming and word reading, seconds	30.0 ± 18.8	26.5 ± 8.5
Word generation fluency	DKEFS letter fluency F + A + S total	40.1 ± 12.9	28.8 ± 3.6^a^
Information processing	Choice reaction task median reaction time, milliseconds	413 ± 38	473 ± 31^a^

Worse cognitive function was seen in soldiers with pituitary dysfunction after blast traumatic brain injury (n = 6) compared to those without pituitary dysfunction (n = 13). Data are expressed as mean ± standard deviation. See Supplementary Methods for further details on cognitive tests.

a *p* < 0.05,

b *p* < 0.005 (unpaired *t* test).

DKEFS = Delis–Kaplan Executive Function System; WASI = Wechsler Abbreviated Scale of Intelligence Similarities and Matrix Reasoning subsets; WTAR = Wechsler Test of Adult Reading.

## Discussion

We have demonstrated a high prevalence of pituitary dysfunction following moderate to severe blast TBI. Almost a third of soldiers with bTBI had anterior pituitary abnormalities, compared to only 2% of age- and gender-matched civilians with moderate to severe nbTBI. The most common pituitary abnormality in bTBI was GH deficiency, followed by hyperprolactinemia, ACTH, and gonadotrophin deficiency. One patient had multiple hormone deficiencies.

We carefully avoided overdiagnosis of pituitary dysfunction. We used identical diagnostic algorithms in the bTBI and nbTBI groups, excluded the presence of macroprolactin, applied strict normal ranges for diagnosing testosterone and TSH deficiency, performed 2 stimulation tests to confirm ACTH or GH deficiencies, and adjusted for the confounds of age and obesity in diagnosing GH deficiency.^22^ This allows us to be confident of our reported prevalence of pituitary dysfunction in both groups.^6^,^7^

Our results suggest that all patients after moderate to severe bTBI should undergo endocrine assessment. Unlike TSH and gonadotrophin deficiency, GH and ACTH deficiency cannot be excluded or always confirmed by basal IGF-I or cortisol measurements. Therefore, dynamic endocrine testing is required. The choice of tests needs to take into account contraindications for use of the ITT, such as seizures, as well as the advantages and disadvantages of each test, including their specificity/sensitivity, age/obesity-adjusted normal ranges, resource implications, local expertise, and drug availability.^7^,^21^,^23^

The presence of pituitary dysfunction after bTBI was not explicable by differences in age, gender, or obesity. The time to endocrine testing was longer in the bTBI than nbTBI group. However, this might be expected to reduce the prevalence of pituitary dysfunction, as it may resolve over time following TBI.^25^ Similarly, use of opiates or other medications does not explain our results. Opiates can have complex neuroendocrine effects, including induction of hypogonadotrophic hypogonadism, and potentially decreasing ACTH secretion but increasing GH secretion.^26^ Although there was greater use of opiates in the bTBI as a whole than in the nbTBI group, the individual pituitary dysfunction seen in each soldier within the bTBI group was not explicable by opiate use. The bTBI group did have more polytrauma than the nbTBI group, which may be a contributory factor, although the mechanism linking peripheral injury to hypothalamic–pituitary dysfunction is uncertain.

Blast appears to produce a distinct pattern of TBI,^15^,^27^ although the mechanism by which blast injury damages the brain remains unclear, limiting our ability to identify those patients at high risk of pituitary dysfunction. The primary blast wave or wind may cause direct injury, or secondary injuries from explosion debris or tertiary injuries from the impact of being thrown by the blast may occur.^28^,^29^ These injuries could affect the hypothalamus, pituitary gland, or pituitary stalk, resulting in damage to cell bodies or white matter connections as well as hypophyseal vessels, local superficial siderosis, inflammation, or hypovolemia/ischemia.

Our imaging results do not provide clear evidence about the precise mechanism of hypothalamic–pituitary damage. We did not see evidence of focal injury to the hypothalamus–pituitary or superficial siderosis, and we excluded bTBI subjects who needed massive blood transfusions. However, pituitary dysfunction may be related to the severity of brain injury after blast exposure, as suggested in nbTBI.^5^ This is supported in our study by the longer duration of PTA in the bTBI than in the nbTBI group (although interpretation may be complicated by sedation and anesthesia), and the presence of more white matter damage^15^ and more skull/facial fractures, and a trend for more cerebral contusions and longer PTA, in those soldiers with than in those without pituitary dysfunction after bTBI. Diffuse axonal injury is common in the corpus callosum after TBI in general,^30^ and posterior fossa white matter tracts are particularly damaged after mild bTBI.^15^ It remains unclear whether the more severe damage to these tracts in bTBI with pituitary dysfunction simply indicates a greater severity of brain injury, or is indicative of a particular injury pattern associated with hypothalamic–pituitary damage.

Our study focused on subjects with a single episode of moderate to severe bTBI. It remains to be determined whether pituitary dysfunction is a significant problem after single, or especially repeated, mild bTBI, because there is evidence that multiple bTBI may augment neurological deficits.^31^ A single previous study has suggested that repeated mild bTBI can produce endocrine disturbance.^32^ However, methodological issues with this study make it difficult to interpret, including their reliance on basal hormone measurements, the definition of normal ranges from a small cohort of control subjects, and the nonstandard assessment of posterior pituitary function.

The trend for worse fatigue, emotional symptoms, social problems, and mood in those soldiers with pituitary dysfunction after bTBI may be related to worse underlying brain injury and/or their endocrine problems. These are well-recognized features of GH deficiency, and lethargy is also seen in cortisol and testosterone deficiency.^8^,^9^,^33^ Similarly, cognitive impairment in soldiers with pituitary dysfunction after bTBI may be related to both greater brain/axonal injury and hormone deficiencies, including GH.^14^,^34^,^35^

Our findings led to substantial changes in clinical management. The soldier with hypogonadotrophic hypogonadism was treated with intramuscular long-acting testosterone. Both soldiers with ACTH deficiency were commenced on hydrocortisone replacement. All 3 soldiers with GH deficiency were >1 year after bTBI and have been started on GH replacement in view of persistent neuropsychological symptoms despite replacement of other pituitary hormones. The soldiers with sufficient follow-up data available have had a symptomatic improvement after 6 months of GH replacement, with adult growth hormone deficiency QoL assessment (AGHDA-QoL) score falling from 19 to 14 (of 25), and Beck Depression Inventory II (BDI-II) score from 36 to 18 (of 63) in 1 subject (M14), and AGHDA-QoL from 14 to 3, and BDI-II falling from 20 to 16 in another (M08) during this period. However, the other soldier receiving GH (M07) is still undergoing dose titration, and so it is too early to assess his symptomatic improvement. The soldiers with mild hyperprolactinemia did not require treatment, as secondary hypogonadism was absent.

In conclusion, this is the first study to demonstrate a high prevalence of anterior pituitary hormone abnormalities after moderate to severe bTBI. The prevalence was greater than in a matched group of civilian nbTBI, suggesting that pituitary dysfunction is a particular problem after blast exposure. Pituitary dysfunction following bTBI was associated with worse cognitive function and greater severity of head injury, including white matter damage. Given that there were no completely diagnostic predictors of pituitary dysfunction in bTBI, we recommend that in clinical practice all soldiers with moderate to severe bTBI undergo routine and comprehensive pituitary function testing during rehabilitation.
