# Supplementary material for: Pituitary Dysfunction after Blast Traumatic Brain Injury: The UK BIOSAP Study
Source: Ann Neurol. 2013 Sep 24;74(4):527–36. doi: 10.1002/ana.23958 (PMC4223931; doi:10.1002/ana.23958)
Supplement: Supplementary file 1 — Supporting Information [file ana0074-0527-SD1.docx]

**Pituitary Dysfunction after Blast Traumatic Brain Injury: UK BIOSAP Study**

David Baxter, David J Sharp, Claire Feeney, Debbie Papadopoulou, Timothy E Ham, Sagar Jilka, Peter J Hellyer, Maneesh C Patel, Alex Bennett, Alan Mistlin, Emer McGilloway, Mark Midwinter, Anthony P Goldstone

**Supplementary Material Annals of Neurology**

**SUPPLEMENTARY FIGURES**

Figure S1. White matter tract regions of interest

Figure S2. Intra-cerebral contusions following blast traumatic brain injury

**SUPPLEMENTARY TABLES**

Table S1. Pituitary-gonadal axis, pituitary-thyroid axis and prolactin in blast traumatic brain injury

Table S2. Growth hormone-IGF-I axis in blast traumatic brain injury

Table S3. ACTH-cortisol axis in blast traumatic brain injury

Table S4. Pituitary dysfunction and structural neuroimaging abnormalities in blast traumatic brain injury

Table S5. Quality of life and symptom questionnaires in non-blast and blast traumatic brain injury

Table S6. Characteristics of soldiers with blast TBI

Table S7. Medications used by soldiers with blast TBI

**SUPPLEMENTARY RESULTS**

Non-pituitary endocrine diagnoses in bTBI and nbTBI cohorts

IGF-I levels in bTBI patients with GH deficiency

Symptoms, quality of life and cognitive function

Interpretation of metyrapone test

**SUPPLEMENTARY METHODS**

Recruitment

Endocrine Testing

Glucagon Stimulation Test

GHRH-Arginine Test

Insulin Tolerance Test (ITT)

Cortisol Day Curve

Metyrapone Stimulation Test

Water Deprivation Test

Neuropsychological Assessments

Structural Imaging

DTI Analysis

**ACKNOWLEDGMENTS**

**SUPPLEMENTARY REFERENCES**

**SUPPLEMENTARY FIGURES**

**Figure S1. White matter tract regions of interest**

**
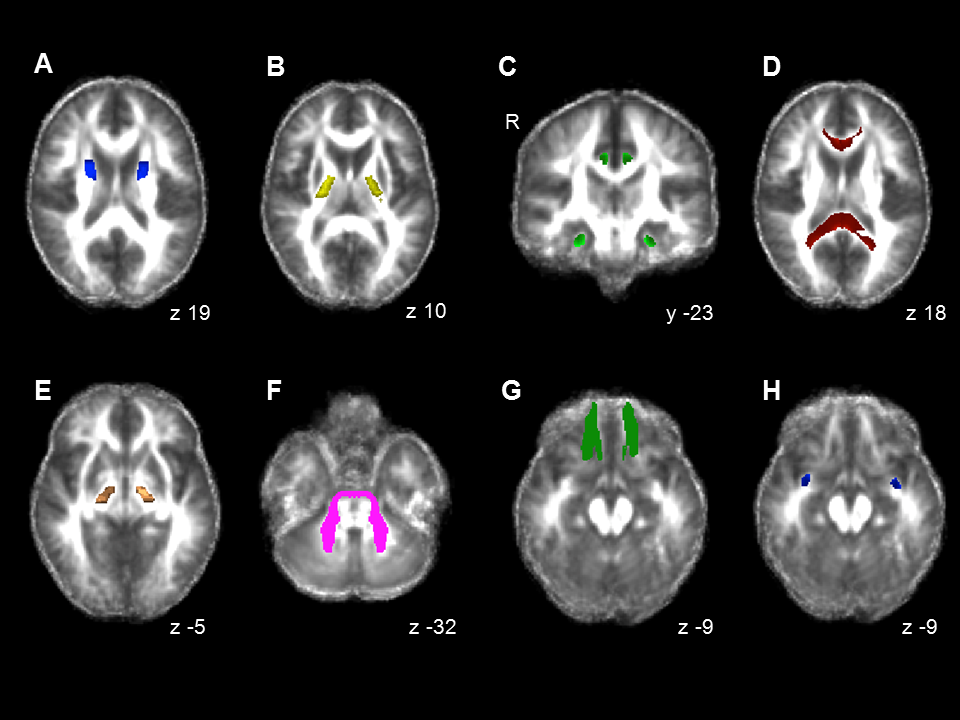
**

Regions of interest (ROIs) used for determination of fractional anisotropy (FA) in soldiers after blast traumatic brain injury (bTBI). Individual color masks overlaid onto group average FA map for soldiers with bTBI (n=19) registered into standard MNI space (using MNI co-ordinates). ROIs are: (A) anterior internal capsule, (B) posterior internal capsule, (C) cingulum, (D) corpus callosum, (E) cerebral peduncles, (F) middle cerebellar peduncles, (G) orbitofrontal white matter, (H) uncinate fasiculi. FA was sampled from areas within a white matter skeleton (not shown) produced by tract based spatial statistics (TBSS).

**Figure S2. Intra-cerebral contusions following blast traumatic brain injury**

**
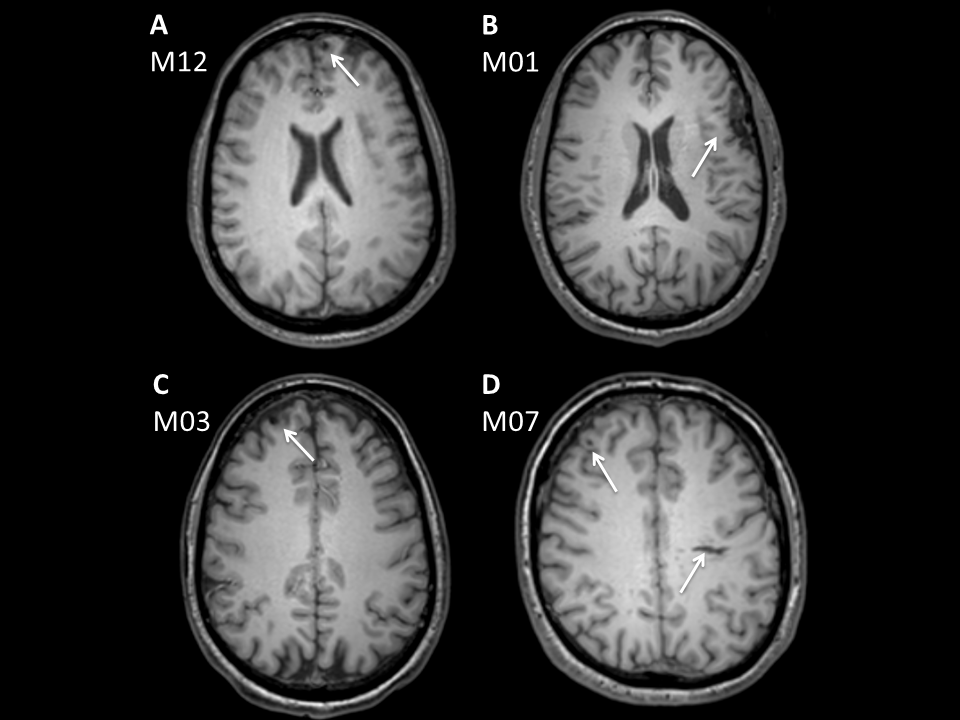
**

High resolution T1 brain scans (axial sections) in subject space showing contusions (arrows) in soldiers after blast TBI (A) without pituitary dysfunction, and (B-D) with pituitary dysfunction. Total contusion volumes for these patients were: (A) 0.2, (B) 9.1, (C) 0.6, (D) 1.0 cm^3^.

**SUPPLEMENTARY TABLES**

**Table S1. Pituitary-gonadal axis, pituitary-thyroid axis and prolactin in blast traumatic brain injury**

Abnormal values indicated by grey shading. * on testosterone replacement, ^#^ calculated from 100 x total testosterone/SHBG, ^a^ to convert to μg/mL divide by 3.467,

^b^ to convert to μg/dL divide by 12.87, ^c^ to convert to μg/dL divide by 15.36, ^d^ remained elevated on repeat measurement with negative macroprolactin. P values from Mann Whitney U test or Fisher’s exact test between groups.

Abbreviations: ACTH: ACTH deficiency, GH: GH deficiency, Gn: Gonadotrophin deficiency, PRL: Hyperprolactinemia

**Table S2. Growth hormone-IGF-I axis in blast traumatic brain injury**

Abnormal values indicated by grey shading. ^a^ to convert to ng/mL divide by 0.131, * using age and BMI normal ranges with BMI 25-30 kg/m^2^ if not calculable due to amputation. P values from Mann Whitney U test between groups. Abbreviations: ACTH: ACTH deficiency, BMI: body mass index, GH: GH deficiency, Gn: Gonadotrophin deficiency, n/a: not applicable, ND: not done, PRL: Hyperprolactinemia.

**Table S3. ACTH-cortisol axis in blast traumatic brain injury**

Abnormal values indicated by grey shading. To convert to μg/dL: divide ^a^ by 27.59, ^b^ by 28.86. P values from Mann Whitney U test between groups. Abbreviations: 11-DOC: 11-deoxycortisol, ACTH: ACTH deficiency, GH: GH deficiency, Gn: Gonadotrophin deficiency, n/a: not applicable, ND: not done, PRL: Hyperprolactinemia.

**Table S4. Pituitary dysfunction and structural neuroimaging abnormalities in blast traumatic brain injury**

Data given as n (%). P values from Fisher’s exact test between groups. Abbreviations: n/a: not applicable, ND: not done.

**Table S5. Quality of life and symptom questionnaires in non-blast and blast traumatic brain injury**

All data expressed as median [interquartile range]. P values from Mann Whitney U test between groups.

Data available in ^a^ n=37, ^b^ n=17, ^c^ n=36, ^d^ n=31, ^e^ n=27, ^f^ n=25, ^g^ n=26.

^h^ excluding subject M12 with undertreated primary hypogonadism

Abbreviations: bTBI: blast TBI, ND: not done, NHP: Nottingham Health Profile, nbTBI: non-blast TBI, SF-36: Short Form 36 Health Survey, TBI: traumatic brain injury.

Note: For AGHDA, BDI-II, Epworth Sleepiness Scale, Pittsburgh Sleep Index and NHP higher score equals worse symptoms and quality of life; for SF-36 lower score equals worse symptoms / quality of life.

**Table S6. Characteristics of soldiers with blast TBI**

All data expressed as median [interquartile range] or n (%). P values from Mann Whitney U test or Fisher’s exact test between groups.

* for analgesia only, # on anti-epilepsy drug

Abbreviations: AIS: Abbreviated Injury Score, BMI: body mass index, GCS: Glasgow Coma Scale, GST: Glucagon stimulation test, ISS: Injury Severity Score, n/a: not available, PTA: Post traumatic amnesia.

**Table S7. Medications used by soldiers with blast TBI**

Abbreviations: MST: morphine sulphate

**SUPPLEMENTARY RESULTS**

*Non-pituitary endocrine diagnoses in bTBI and nbTBI cohorts*

Other non-pituitary endocrine disorders were diagnosed in both groups. Primary hypogonadism due to perineum/testicular blast injury had been found in 4 out of 19 soldiers (21.2%), none of whom had pituitary dysfunction (Table 2 and S1). Although at the time of our assessment all these subjects were already on testosterone replacement, 3 had documented increased gonadotophins before its initiation (Table S1). One of these (M12) was under-replaced with testosterone at the time of assessment. A high prevalence of perineal blast injury has previously been reported in soldiers exposed to IED (Mossadegh et al., 2012). One control patient with nbTBI had a pre-existing diagnosis of primary hypothyroidism, and another had previously undiagnosed primary hypogonadism of unknown cause unrelated to their nbTBI.

*IGF-I levels in bTBI patients with GH deficiency*

IGF-I levels were within the normal range in all those soldiers with GH deficiency. When comparing those soldiers with bTBI who had GH deficiency (n=3) to those without GH deficiency (n=16), absolute IGF-I levels tended to be lower in those with than without GH deficiency (median [IQR] 18.2 [16.7-22.3] vs. 27.1 (19.9-31.6], P=0.11). However IGF-I relative to median of age-related reference range were similar between groups (0.66 [0.60-0.73] vs. 0.79 [0.63-1.00], P=0.40) (Table S1).

S*ymptoms, quality of life and cognitive function*

In our cohort of soldiers with bTBI, subjective symptoms included worsening of their memory (70%), changes in mood (70%), difficulty concentrating (65%), difficulty sleeping (55%), headaches (45%), and dizziness (30%).

Consistent with their higher prevalence of polytrauma and amputations, the soldiers with bTBI had significantly worse scores for physical activity (P=0.02) and daily living problems (P=0.04) from the Nottingham Health Profile (NHP) questionnaire, with a tendency for worse NHP pain scores (P=0.08) and change in health from the Short Form-36 (SF-36) quality of life questionnaire (P=0.06), than the control nbTBI group (Table S5). However there were no significant differences in measures of depression and emotional well-being (from Beck Depression Inventory-II), NHP and SF-36 questionnaires) between the bTBI and nbTBI groups (P=0.30-0.71) (Table S5).

In the bTBI group, soldiers with pituitary dysfunction had trends towards worse measures of QoL and symptom scores in several domains compared to those without pituitary dysfunction (Table S5). Soldiers after bTBI with pituitary dysfunction had trends for higher AGHDA QoL score (P=0.10), worse scores for emotional reactions (NHP, P=0.10), social isolation (NHP, P=0.13), role limitations due to physical health (SF-36, P=0.10), energy/fatigue (SF-36, P=0.15), and social functioning (SF-36, P=0.18), and higher depression scores (BDI-II, P=0.10), though none had symptoms suggesting severe depression (all scores <28/63).

*Interpretation of metyrapone test*

Although the metyrapone test is not a commonly used test for ACTH deficiency (Grossman 2010), it was only needed for the confirmatory diagnosis in one soldier (M03). Furthermore that subject also had very low cortisol levels throughout their day curve ≤50 nmol/L (≤1.81 μg/dL) confirming the diagnosis of ACTH deficiency. The second soldier with ACTH deficiency (M10) failed their cortisol response to insulin-induced hypoglycemia (peak 268 nmol/L), and also had low cortisol levels (<100 nmol/L, <3.62 μg/dL) at 1200h on their day curve supporting the diagnosis. Other soldiers who initially had low cortisol responses to glucagon stimulation, subsequently had ACTH deficiency excluded on the basis of normal responses to ITT (M02) or Metyrapone test (M10), but both also had subsequent high basal morning cortisol levels (M02, M10, >400 nmol/L, 14.50 μg/dL).

Previous studies comparing the metyrapone test to more commonly used tests for ACTH deficiency have demonstrated the metyrapone test to have specificity, sensitivity and concordance (accuracy) rates of 77-100%, 64-89%, 74-84% (n=17-32) and 86, 91, 87% (n=87) with the ITT and ACTH stimulation test respectively (Fiad et al. 1994; Courtney et al. 2000; Giordano et al. 2008). Furthermore in a recent audit of patients from our endocrine clinics suspected of having ACTH deficiency (n=24, excluding soldiers with bTBI from this study), we have found an overall 92% concordance rate between results of a metyrapone test, and the ACTH stimulation test (n=12, normal response >480 nmol/L or 17.40 μg/dL, using alignment of the previous 550 nmol/L cut-off to the new Architect i2000 assay) or ITT (n=13) (unpublished observations). In this analysis, all patients failing the metyrapone test (n=5) also failed an ITT. The overall specificity for the metyrapone test in diagnosing ACTH deficiency was 100% and sensitivity was 71% (unpublished observations).

**SUPPLEMENTARY METHODS**

***Recruitment***

Ethical approval was granted by the Ealing and West London Hospitals Research Ethics Committee. Studies were performed according to the Declaration of Helsinki and all soldiers gave informed written consent.

Inclusion of a military combat nbTBI group would have been a useful in addition to the civilian nbTBI group to control for active military service in an identical theatre. However in UK soldiers experiencing nbTBI in Afghanistan, the majority are due to gunshot wounds that are either fatal or complicated by penetrating brain injury often requiring surgery. The lower prevalence of military non-penetrating nbTBI, primarily due to road traffic accidents, precluded endocrine assessment of a sufficient number of such soldiers to be included in this study.

Both bTBI and nbTBI subjects had clinical assessment, calculation of their Abbreviated Injury Scores (AIS) for each body region including brain, and total Injury Severity Score (ISS) (Baker et al. 1974; Hawley 1996), and completed quality of life (QoL) and symptom questionnaires: Assessment of Growth Hormone Deficiency in Adults (QoL-AGHDA); Beck Depression Inventory-II (BDI-II); Nottingham Health Profile (NHP); Short Form 36 Health Survey (SF-36), Pittsburgh Sleep Quality Index and Epworth Sleepiness Scale (Hunt et al. 1985; Buysse et al. 1989; Johns 1991; Ware & Sherbourne 1992; Beck et al. 1996; McKenna et al. 1999). Soldiers were excluded if they had needed massive blood transfusion so as to exclude pituitary dysfunction secondary to hypovolemic shock (Stainsby et al. 2006).

***Endocrine Testing***

Endocrine assessment included baseline measurement of serum anterior pituitary hormones: TSH, free T4, free T3, prolactin, FSH, LH, testosterone (Abbott Architect Ci8200), ACTH, cortisol, GH, IGF-I (Immulite® 2000) and sex hormone binding globulin (SHBG). Free androgen index was calculated as 100 x total testosterone / SHBG.

A diagnosis of hyperprolactinemia was made on the basis of two consecutively raised prolactin readings (above upper reference range, Table 1) and a negative macroprolactin, an immunological artefact leading to misdiagnosis of hyperprolactinemia (assessed by PEG precipitation) (Smith et al. 2007). Subjects who met these criteria had MRI of the pituitary including gadolinium contrast to rule out an incidental pituitary tumour.

A diagnosis of gonadotrophin deficiency was made on the basis of a low morning testosterone <10 nmol/L (<2.9 ng/mL) with low or non-elevated LH (NR 1.7-12.0 IU/L) and FSH (NR 1.7-8.0 IU/L). If sex hormone binding globulin (SHBG) was low (<15 nmol/L), then FAI needed to be <30 for the diagnosis. Primary hypogonadism was defined as a low morning testosterone or FAI with elevated FSH and/or LH.

Growth hormone (GH) deficiency was defined as failure on 2 dynamic endocrine tests performed in the morning: (i) Glucagon Stimulation Test (GST) used as initial screening test and (ii) a confirmatory 2^nd^ line test, either the GHRH-Arginine Test or an Insulin Tolerance Test (ITT).

Similarly, a diagnosis of ACTH deficiency was made on the basis of failure on 2 dynamic endocrine tests performed in the morning: (i) a GST, and (ii) an ITT or an overnight Metyrapone Stimulation Test (MST). A 5 point Cortisol Day Curve (CDC) was also used to help confirm or exclude ACTH deficiency, and assess the need for maintenance hydrocortisone replacement as opposed to just during intercurrent illness.

An ITT was not routinely performed because of the prevalence of relative and absolute contraindications in this population. In our cohort 10.5% of soldiers after bTBI and 10.3% of controls after nbTBI had an absolute contraindication (history of seizures, ischemic heart disease, cardiac arrhythmias, abnormal ECG), whilst an additional 21.1% and 53.8% had a relative contraindication (intra-cerebral contusion, intra-cranial hemorrhage). If further confirmatory testing was required because of equivocal findings on the second dynamic test (e.g. difficulty calculating BMI in soldiers with amputations), and no contraindications were present, an ITT was carried out in addition to the glucagon test and GHRH-Arginine or metyrapone test.

Diabetes insipidus was screened for on the basis of symptoms (polyuria and polydipsia) and measurement of paired random clinic urine and plasma osmolalities. If clinically indicated, a Water Deprivation Test was performed (n=6 controls with nbTBI, n=1 soldier with bTBI).

All dynamic endocrine tests were carried out in an in-patient facility at Charing Cross Hospital, London or St. Mary’s Hospital, London. A summary of the algorithm used to define pituitary dysfunction is shown in Table 1.

***Glucagon Stimulation Test (GST)***

Following an overnight fast, patients had basal blood samples. Glucagon (GlucaGen™, Novo Nordisk Pharmaceuticals, Crawley, UK 1 mg, or 1.5 mg if weight >90 kg) was administered intramuscularly. Blood samples for glucose, serum cortisol and GH were taken at 90, 120, 150 and 180 minutes after glucagon administration from an intravenous (IV) cannula. The majority of subjects (89% soldiers and 70% controls) also had samples taken at 210 and 240 minutes. An abnormal response was defined as a peak GH <5 μg/L and cortisol <350 nmol/L (<12.7 μg/dL) during the test (Yuen et al. 2009; Cegla et al. 2012). Subjects who failed to reach these thresholds underwent at least one additional confirmatory dynamic test.

The method for cortisol determination was changed in August 2010 from the Immulite® 2000 assay (Siemens) to a chemiluminescence immunoassay with the Architect i2000 (Abbott, UK). To assure comparability, quality controls and linear regression analysis were performed (data not shown) and results from the Immulite assay were aligned with the Architect i2000 assay. The Architect assay has coefficients of variation <10% for cortisol levels of 83–967 nmol/L (3.0-35.0 μg/dL).

***GHRH-Arginine Test***

Following an overnight fast, patients had blood samples taken for GH and IGF-I measurement at 0 minutes. GHRH (Somatorelin, Ferring) 1μg/kg was given as a bolus IV injection into one arm followed by the IV infusion of 0.5g/kg L-arginine monohydrochloride (Stockport Pharmaceuticals) as a 10% solution (30g/300mL up to a maximum of 30g) in normal saline over 30 minutes (Colao et al., 2009). Further blood samples for GH estimation were taken at +30, 60, 90, 120 and 150 minutes after the start of the arginine infusion.

GH cut offs to confirm GH deficiency varied according to age and BMI. For age groups 15-25 years old, 26-65 years old and older than 65 years, GH cut-offs were respectively <15.6, <11.7, and <8.5 μg/L, <11.8, <8.1, and <5.5 μg/L, and <9.2, <6.1, and <4.0 μg/L, respectively, in lean (BMI<25.0kg/m^2^), overweight (BMI 25.0-30.0 kg/m^2^) and obese (BMI>30.0 kg/m^2^) subjects (Colao et al. 2009). If amputations precluded accurate determination of BMI then cut-offs in the overweight range were used.

***Insulin Tolerance Test (ITT)***

Following an overnight fast, basal blood samples were taken and IV insulin Actrapid (NovoNordisk) administered (0.15 U/kg). Blood samples were taken for GH, cortisol and glucose at 0, 30, 60, 90, and 120 mins. Blood glucose was also measured simultaneously. Once adequate hypoglycemia (<2.2 mmol/L, <39.6 mg/dL) was achieved, hypoglycemia was reversed with oral glucose and at least two further blood specimens were taken before test completion.

Abnormal cortisol response was defined as peak cortisol of <450 nmol/L (<16.3 μg/dL) providing adequate hypoglycemia was achieved (using alignment of the previous 500 nmol/L cut-off to the new Architect i2000 assay). Severe GH deficiency was defined as a peak GH <3 μg/L (Plumpton & Besser 1969; Fish et al. 1986; Molitch et al. 2011).

***Cortisol Day Curve***

Blood samples were taken from an IV cannula for serum cortisol estimation at 0900h, 1200h, 1500h, 1800h and 2100h (Immulite ® 2000 assay (Siemens) or Architect i2000 (Abbott, UK), and plasma ACTH at 0900h. Results helped confirm (cortisol <100 nmol/L or 3.62 μg/dL at 0900 or 1200h), or exclude (cortisol >400 nmol/L or 14.50 μg/dL at 0900h) ACTH deficiency, and assess the need for maintenance hydrocortisone replacement as opposed to just during intercurrent illness (Grossman 2010).

***Metyrapone Stimulation Test***

Patients were given oral metyrapone (Metopirone™, Alliance Pharmaceuticals, Chippenham, UK) (30 mg/kg), at midnight with a snack, according to their body weight (<70 kg 2.0g, 70-90 kg 2.5g, >90kg 3.0g) (Steiner et al. 1994; Cegla et al. 2012). At 0900h the following morning, blood samples were taken for serum cortisol, 11-DOC (Biosource, Oxford Biosystems, UK) and plasma ACTH (Immulite® 2000, Siemens). Hydrocortisone 10 mg was given orally to counteract hypocortisolism and the patients were discharged.

Metyrapone causes inhibition of 11 β-hydroxylase (used in the conversion of 11-deoxycortisol to cortisol) and cortisol suppression to <200 nmol/L (7.25 μg/dL) is the desired threshold to stimulate ACTH drive. Subjects were considered to be ACTH sufficient if 11-DOC was >200 nmol/L or, if the 11-DOC was unavailable, if ACTH >60 ng/L (Steiner et al. 1994; Cegla et al. 2012).

***Water Deprivation Test***

This was carried out in two stages on non-fasted subjects (Vokes & Robertson 1988).

In Stage 1, patients drank no fluid from 0830-1630h. Weight and urine volume (after urine passed and discarded at t=0) were recorded hourly. The test was stopped if >3% weight was lost. Urine specimens were taken for osmolality from the total hourly sample passes over 0830-0930h (U1), 1130-1230h (U2), 1430-1530h (U3), 1530-1630h (U4). Blood samples were taken for osmolality and plasma sodium at 0900h (P1), 1200h (P2), 1500h (P3) and 1600h (P4).

In Stage 2, at 1630 hrs following the dehydration stage, Desmopressin (DDAVP 2μg IM or 20μg intra-nasally) was administered. Urine volumes were recorded and urine specimens for osmolality measurement were taken every hour until test completion at 2030h.

Central diabetes insipidus was defined as plasma concentration to >300 mosmol/kg with inappropriately hypotonic urine (U3:P3 or U4:P4 ≤1.9) or urine osmolality <350 mosmol/kg. In addition, urine was required to concentrate to >150% of previous highest value following DDAVP administration.

***Neuropsychological Assessments***

Each soldier completed a standardized neuropsychological test battery previously shown to be sensitive to cognitive impairment associated with traumatic brain injury (Kinnunen et al. 2011). The cognitive functions of specific interest were indexed by: (i) current verbal and non-verbal reasoning ability via the Wechsler Abbreviated Scale of Intelligence Similarities and Matrix Reasoning subtests (Wechsler 1999); (ii) associative learning and memory via the immediate recall score on the People Test from the Doors and People Test (Baddeley 2011); (iii) the executive functions of set-shifting, inhibitory control, cognitive flexibility and word generation fluency via the Trail Making Test alternating-switch cost index (time to complete alternating letter and number Trails B - time to complete numbers only Trail A) and two indices from the Delis-Kaplan Executive Function System (Reitan 1958; Delis et al. 2001), namely the inhibition/switching minus baseline score from the Colour-Word subtest (high scores indicating poor performance) and the total score on Letter Fluency; and (iv) information processing speed via the median reaction time for accurate responses on a simple computerized choice reaction task (Kinnunen et al. 2011). The Wechsler Test of Adult Reading (WTAR) was also administered as a measure of pre-morbid intelligence (Green et al. 2008).

***Structural Imaging***

Each soldier had standard high-resolution T1 and gradient-echo (T2*) (1.75x1.75x2mm^3^) imaging to assess focal brain injury and evidence of microbleeds, superficial siderosis, presence and location of contusions and gross pituitary injury. All structural MR scans were reviewed by a single experienced consultant neuroradiologist. Contusion volume was calculated by converting the T1 images into standard 1mm MNI brain space using FLIRT (FMRIB, University of Oxford, UK) and manually drawing a mask in the z plane.

MRI was performed on 3T Achieva scanner (Philips Medical Systems, Netherlands) using an 8 channel head coil. The T1 and T2*-weighted images were obtained prior to DTI. For DTI, diffusion-weighted volumes with gradients applied in 16 non-collinear directions were collected in each of the four DTI runs, resulting in a total of 64 directions. The following parameters were used: 73 contiguous slices, slice thickness 2mm, field of view 224mm, matrix 128 x 128 (voxel size 1.75x1.75x2 mm^3^), b value 1000 and four images with no diffusion weighting (b=0s/mm^2^).

The images were registered to the b0 image by affine transformations to minimize distortion due to motion and eddy currents and then brain-extracted using Brain Extraction Tool (Smith 2002) from the FMRIB Software Library image processing toolbox (Smith et al. 2004; Woolrich et al. 2009). Fractional anisotropy (FA) maps were generated using the Diffusion Toolbox (Behrens et al. 2003).

***DTI Analysis***

DTI analysis used TBSS and non-parametric permutation based statistics for whole brain and region of interest (ROI) analysis (FMRIB software, FSL, University of Oxford, UK).

Voxelwise analysis of the fractional anisotropy, was carried out using TBSS in the FMRIB Software Library (Smith et al. 2004; Smith et al. 2006). Image analysis using TBSS involved a number of steps: (i) non-linear alignment of all subjects’ FA images into common FMRIB58 FA template space; (ii) affine-transformation of the aligned images into standard MNI152 1mm space; (iii) averaging of the aligned FA images to create a 4D mean FA image; (iv) thinning of the mean FA image to create a mean FA ‘skeleton’ representing the centre of all white matter tracts, and in this way removing partial-volume confounds; and (v) thresholding of the FA skeleton at FA 0.2 to suppress areas of extremely low mean FA and exclude those with considerable inter-individual variability. Non-parametric permutation-based statistics were employed using randomize with threshold-free cluster enhancement and 5000 permutations (Nichols & Holmes 2002; Smith & Nichols 2009). A threshold of P<0.05 was then applied on the results, corrected for multiple comparisons. Age was included as a covariate of no interest in all TBSS analyses.

Regions of interest (ROI) were defined using the John Hopkins University (JHU) white matter atlas. We chose 10 areas that represented white matter regions throughout the whole brain and have been shown to be damaged in nbTBI as well as mild bTBI (Kinnunen et al. 2011; MacDonald et al. 2011). These regions were: anterior and posterior internal capsules, cingulum, body/genu and splenium of the corpus callosum, cerebral peduncles, middle cerebellar peduncles, and uncinate fasciuli (Fig. S1). In addition a cerebellum ROI mask was drawn manually and an orbitofrontal white matter ROI mask made using the Washington University, St Louis criteria from the standard MNI152 1mm T1 brain (MacDonald et al. 2011). A repeated measures ANOVA was performed to assess the overall significance effect of pituitary dysfunction on FA, including group, ROI and group x ROI interaction as independent variables, with post-hoc 2-tailed t-tests for comparison of FA in individual ROIs between groups.

**SUPPLEMENTARY REFERENCES**

Baddeley A. (2011) Doors and people test: a test of visual and verbal recall and recognition. Thames Valley Test Company, Bury St Edmunds, Suffolk, UK.

Baker SP, O'Neill B, Haddon W, Jr., Long WB. (1974) The injury severity score: a method for describing patients with multiple injuries and evaluating emergency care. J Trauma 14: 187-96.

Beck AT, Steer RA, Ball R, Ranieri W. (1996) Comparison of Beck Depression Inventories -IA and -II in psychiatric outpatients. J Pers Assess 67: 588-97.

Behrens TE, Woolrich MW, Jenkinson M, Johansen-Berg H, Nunes RG, Clare S, Matthews PM, Brady JM, Smith SM. (2003) Characterization and propagation of uncertainty in diffusion-weighted MR imaging. Magn Reson Med 50: 1077-88.

Buysse DJ, Reynolds CF, Monk TH, Berman SR, Kupfer DJ. (1989) The Pittsburgh Sleep Quality Index: a new instrument for psychiatric practice and research. Psychiatry Res 28: 193-213.

Cegla J, Jones B, Seyani L, Papadoulou D, Wynne K, Martin NM, Meeran K, Chapman R, Donaldson M, Goldstone AP, Tan T. (2012) Comparison of the overnight metyrapone and glucagon stimulation tests in the assessment of secondary hypoadrenalism. Clin Endocrinol (Oxf) doi: 10.1111/cen.12043.

Colao A, Di SC, Savastano S, Rota F, Savanelli MC, Aimaretti G, Lombardi G. (2009) A reappraisal of diagnosing GH deficiency in adults: role of gender, age, waist circumference, and body mass index. J Clin Endocrinol Metab 94: 4414-22.

Courtney CH, McAllister AS, McCance DR, Hadden DR, Leslie H, Sheridan B, Atkinson AB. (2000) The insulin hypoglycaemia and overnight metyrapone tests in the assessment of the hypothalamic-pituitary-adrenal axis following pituitary surgery. Clin Endocrinol (Oxf) 53: 309-12.

Delis DC, Kaplan E, Kramer JH. (2001) Delis-Kaplan Executive Function System. The Psychological Corporation, San Antonio, Texas, USA.

Fiad TM, Kirby JM, Cunningham SK, McKenna TJ. (1994) The overnight single-dose metyrapone test is a simple and reliable index of the hypothalamic-pituitary-adrenal axis. Clin Endocrinol (Oxf) 40: 603-9.

Fish HR, Chernow B, O'Brian JT. (1986) Endocrine and neurophysiologic responses of the pituitary to insulin-induced hypoglycemia: a review. Metabolism 35: 763-80.

Giordano R, Picu A, Bonelli L, Balbo M, Berardelli R, Marinazzo E, Corneli G, Ghigo E, Arvat E. (2008) Hypothalamus-pituitary-adrenal axis evaluation in patients with hypothalamo-pituitary disorders: comparison of different provocative tests. Clin Endocrinol (Oxf) 68: 935-41.

Green RE, Melo B, Christensen B, Ngo LA, Monette G, Bradbury C. (2008) Measuring premorbid IQ in traumatic brain injury: an examination of the validity of the Wechsler Test of Adult Reading (WTAR). J Clin Exp Neuropsychol 30: 163-72.

Grossman AB. (2010) Clinical Review: The diagnosis and management of central hypoadrenalism. J Clin Endocrinol Metab 95: 4855-63.

Hawley A. (1996) Trauma management on the battlefield: a modern approach. J R Army Med Corps 142: 120-5.

Hunt SM, McEwen J, McKenna SP. (1985) Measuring health status: a new tool for clinicians and epidemiologists. J R Coll Gen Pract 35: 185-8.

Johns MW. (1991) A new method for measuring daytime sleepiness: the Epworth sleepiness scale. Sleep 14: 540-5.

Kinnunen KM, Greenwood R, Powell JH, Leech R, Hawkins PC, Bonnelle V, Patel MC, Counsell SJ, Sharp DJ. (2011) White matter damage and cognitive impairment after traumatic brain injury. Brain 134: 449-63.

MacDonald CL, Johnson AM, Cooper D, Nelson EC, Werner NJ, Shimony JS, Snyder AZ, Raichle ME, Witherow JR, Fang R, Flaherty SF, Brody DL. (2011) Detection of blast-related traumatic brain injury in U.S. military personnel. N Engl J Med 364: 2091-100.

McKenna SP, Doward LC, Alonso J, Kohlmann T, Niero M, Prieto L, Wiren L. (1999) The QoL-AGHDA: an instrument for the assessment of quality of life in adults with growth hormone deficiency. Qual Life Res 8: 373-83.

Molitch ME, Clemmons DR, Malozowski S, Merriam GR, Vance ML. (2011) Evaluation and treatment of adult growth hormone deficiency: an Endocrine Society clinical practice guideline. J Clin Endocrinol Metab 96: 1587-609.

Nichols TE, Holmes AP. (2002) Nonparametric permutation tests for functional neuroimaging: a primer with examples. Hum Brain Mapp 15: 1-25.

Plumpton FS, Besser GM. (1969) The adrenocortical response to surgery and insulin-induced hypoglycaemia in corticosteroid-treated and normal subjects. Br J Surg 56: 216-9.

Reitan R. (1958) The validity of the Trail Making Test as an indicator of organic brain damage. Percept Mot Skills 8: 276.

Smith SM. (2002) Fast robust automated brain extraction. Hum Brain Mapp 17: 143-55.

Smith SM, Jenkinson M, Johansen-Berg H, Rueckert D, Nichols TE, Mackay CE, Watkins KE, Ciccarelli O, Cader MZ, Matthews PM, Behrens TE. (2006) Tract-based spatial statistics: voxelwise analysis of multi-subject diffusion data. Neuroimage 31: 1487-505.

Smith SM, Jenkinson M, Woolrich MW, Beckmann CF, Behrens TE, Johansen-Berg H, Bannister PR, De LM, Drobnjak I, Flitney DE, Niazy RK, Saunders J, Vickers J, Zhang Y, De SN, Brady JM, Matthews PM. (2004) Advances in functional and structural MR image analysis and implementation as FSL. Neuroimage 23 Suppl 1: S208-S219.

Smith SM, Nichols TE. (2009) Threshold-free cluster enhancement: addressing problems of smoothing, threshold dependence and localisation in cluster inference. Neuroimage 44: 83-98.

Smith TP, Kavanagh L, Healy ML, McKenna TJ. (2007) Technology insight: measuring prolactin in clinical samples. Nat Clin Pract Endocrinol Metab 3: 279-89.

Stainsby D, MacLennan S, Thomas D, Isaac J, Hamilton PJ. (2006) Guidelines on the management of massive blood loss. Br J Haematol 135: 634-41.

Steiner H, Bahr V, Exner P, Oelkers PW. (1994) Pituitary function tests: comparison of ACTH and 11-deoxy-cortisol responses in the metyrapone test and with the insulin hypoglycemia test. Exp Clin Endocrinol 102: 33-8.

Vokes TJ, Robertson GL. (1988) Disorders of antidiuretic hormone. Endocrinol Metab Clin North Am 17: 281-99.

Ware JE, Sherbourne CD. (1992) The MOS 36-item short-form health survey (SF-36). I. Conceptual framework and item selection. Med Care 30: 473-83.

Wechsler D. (1999) WASI: Wechsler Abbreviated Scale of Intelligence. The Psychological Corporation, San Antonio, Texas, USA.

Woolrich MW, Jbabdi S, Patenaude B, Chappell M, Makni S, Behrens T, Beckmann C, Jenkinson M, Smith SM. (2009) Bayesian analysis of neuroimaging data in FSL. Neuroimage 45 Suppl 1: S173-S186.

Yuen KC, Biller BM, Molitch ME, Cook DM. (2009) Clinical review: Is lack of recombinant growth hormone (GH)-releasing hormone in the United States a setback or time to consider glucagon testing for adult GH deficiency? J Clin Endocrinol Metab 94: 2702-7.
